# The Importance of Poly(ADP-Ribose) Polymerase as a Sensor of Unligated Okazaki Fragments during DNA Replication

**DOI:** 10.1016/j.molcel.2018.06.004

**Published:** 2018-07-19

**Authors:** Hana Hanzlikova, Ilona Kalasova, Annie A. Demin, Lewis E. Pennicott, Zuzana Cihlarova, Keith W. Caldecott

**Affiliations:** 1 Genome Damage and Stability Centre & Sussex Drug Discovery Centre, School of Life Sciences, University of Sussex, Falmer, Brighton BN1 9RQ, UK; 2 Department of Genome Dynamics, Institute of Molecular Genetics of the ASCR, v.v.i., 142 20 Prague 4, Czech Republic

**Keywords:** DNA replication, DNA repair, DNA strand break, PARP1, postreplication repair

## Abstract

Poly(ADP-ribose) is synthesized by PARP enzymes during the repair of stochastic DNA breaks. Surprisingly, however, we show that most if not all endogenous poly(ADP-ribose) is detected in normal S phase cells at sites of DNA replication. This S phase poly(ADP-ribose) does not result from damaged or misincorporated nucleotides or from DNA replication stress. Rather, perturbation of the DNA replication proteins LIG1 or FEN1 increases S phase poly(ADP-ribose) more than 10-fold, implicating unligated Okazaki fragments as the source of S phase PARP activity. Indeed, S phase PARP activity is ablated by suppressing Okazaki fragment formation with emetine, a DNA replication inhibitor that selectively inhibits lagging strand synthesis. Importantly, PARP activation during DNA replication recruits the single-strand break repair protein XRCC1, and human cells lacking PARP activity and/or XRCC1 are hypersensitive to FEN1 perturbation. Collectively, our data indicate that PARP1 is a sensor of unligated Okazaki fragments during DNA replication and facilitates their repair.

## Highlights

• Endogenous PAR is detected during S phase at sites of DNA replication
• S phase PAR is increased by perturbing enzymes that process Okazaki fragments
• PARP activity during S phase is prevented by suppressing Okazaki fragment formation
• S phase PAR recruits XRCC1 and protects cells from the effect of FEN1 inhibitor

## Graphical Abstract

**Figure:**
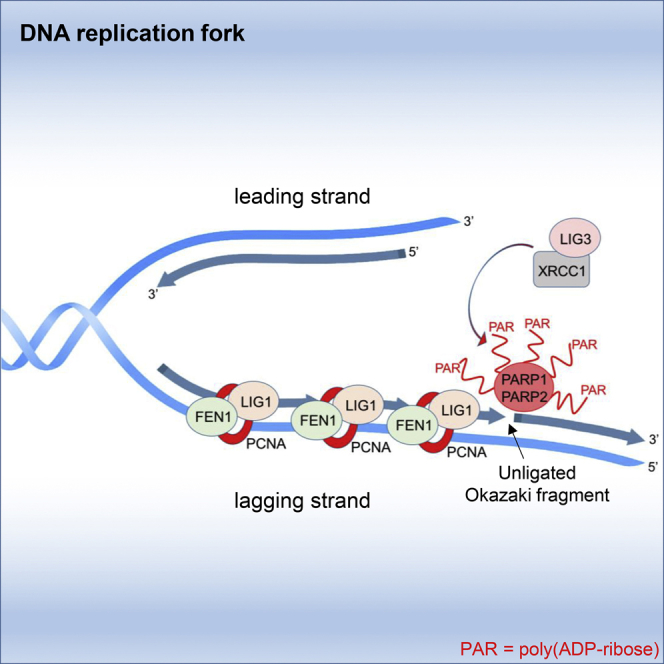

## Teaser

Hanzlikova et al. propose that PARP1 is a sensor of incompletely processed replication intermediates and suggest that, during normal S phase, a sub-fraction of Okazaki fragments escapes processing by the canonical FEN1 and LIG1 pathway, resulting in a collection of DNA single-strand nicks, gaps, and/or flaps that require processing by a PARP1 and XRCC1-dependent “backup” pathway.

## Introduction

ADP-ribosyl transferases (ADPRTs) comprise a superfamily of enzymes that post-translationally modify themselves and/or other proteins with mono- or poly(ADP-ribose) (Amé et al., 2004, Hottiger et al., 2010). The archetypal member of this family is poly(ADP-ribose) polymerase-1 (PARP1, also known as ADPRT1), an abundant nuclear enzyme that regulates multiple cellular processes, including transcription, chromatin remodeling, and DNA damage signaling. With respect to DNA damage signaling, PARP1 binds to and is activated by both DNA single-strand breaks (SSBs) and DNA double-strand breaks (DSBs), serving as a rapid and sensitive cellular sensor of DNA breakage (Benjamin and Gill, 1980, Ikejima et al., 1990). In addition to PARP1, PARP2 and PARP3 are also activated by binding to DNA strand breaks (Amé et al., 1999, Grundy et al., 2016, Langelier et al., 2014, Rulten et al., 2011). However, to date, only PARP1 and PARP2 activity has been detected in cells, perhaps because PARP3 modifies proteins primarily with mono(ADP-ribose), whereas PARP1 and PARP2 frequently modify proteins with poly(ADP-ribose). PARP1 accounts for more than 80% of poly(ADP-ribose) synthesis, with PARP2 accounting for the remainder (Amé et al., 1999, Hanzlikova et al., 2017).

A number of roles for PARP signaling at sites of DNA damage have been identified following exogenous genotoxic stress. For example, ADP-ribosylation can facilitate chromatin relaxation at DNA breaks, either directly via ADP-ribose-mediated charge repulsion or indirectly by recruitment of chromatin modifiers such as ALC1 and aprataxin and PNKP-like factor (APLF) (Ahel et al., 2009, Mehrotra et al., 2011, Poirier et al., 1982, Singh et al., 2017, Timinszky et al., 2009). In addition, poly(ADP-ribose) synthesis at sites of DNA replication fork stalling or damage induced by DNA replication inhibitors can regulate Chk1 protein kinase, Mre11 nuclease, and RECQ helicase activities, regulating replication fork resection, degradation, and restart (Berti et al., 2013, Bryant et al., 2009, Ding et al., 2016, Min et al., 2013, Ray Chaudhuri et al., 2012, Sugimura et al., 2008, Yang et al., 2004). Among the commonest DNA damage structures induced by genotoxins that activate PARP1 and PARP2 are SSBs, which arise both from direct attack of the sugar phosphate backbone by reactive oxygen species or topoisomerase enzymes and indirectly as obligate intermediates of several different DNA excision repair processes (Caldecott, 2008). PARP signaling at SSBs recruits proteins that facilitate SSB repair (SSBR), the most studied of which is X-ray repair cross-complementing protein 1 (XRCC1). XRCC1 is a scaffold protein that accelerates SSBR by recruiting, and in some cases stabilizing and/or stimulating, the enzymes with which it interacts (Caldecott et al., 1994, Caldecott et al., 1995, Loizou et al., 2004, Whitehouse et al., 2001). PARP1 and/or PARP2 signaling recruits XRCC1 protein complexes via a direct interaction between poly(ADP-ribose) and a BRCA1 C-terminal (BRCT) domain in XRCC1 (Breslin et al., 2015, Caldecott et al., 1996, El-Khamisy et al., 2003, Hanzlikova et al., 2017, Masson et al., 1998, Okano et al., 2003).

Given the multiple roles and importance of PARP1 and PARP2 following exogenous genotoxic stress, it is important to identify which sources of poly(ADP-ribose) synthesis predominate in unperturbed cells and the functional significance of this signaling. That PARP1 and PARP2 fulfil important roles in DNA metabolism in unperturbed cells is suggested by several observations. First, mice lacking both *Parp1*^*−/−*^ and *Parp2*^*−/−*^ exhibit embryonic lethality and fail to develop beyond embryonic day 7.0 (E7.0–E8.0), most likely because of problems arising during the rapid cycles of DNA replication within the epiblast during gastrulation (Ménissier-de Murcia et al., 2003). Second, small-molecule inhibitors of PARP enzymes invoke synthetic lethality in cells in which homologous recombination (HR)-mediated repair is attenuated, a feature that has been exploited in the clinic to selectively kill *BRCA1*- and *BRCA2*-mutated cancer cells (Bryant et al., 2005, Farmer et al., 2005). It has been suggested that HR proteins are necessary in the presence of PARP inhibitors to repair and/or regulate stalled or damaged replication forks, but the endogenous DNA lesions and/or structures that are trapped by PARP inhibitors to trigger DNA replication fork damage are unclear. A major problem in identifying sites of endogenous PARP activity has been the difficulty to detect endogenous sites of poly(ADP-ribose) synthesis in the absence of exogenous genotoxic stress. Here we have circumvented this problem by employing short incubations with an inhibitor of poly(ADP-ribose) glycohydrolase (PARG), the enzyme primarily responsible for poly(ADP-ribose) catabolism (Davidovic et al., 2001, Lin et al., 1997, Slade et al., 2011). Strikingly, we show that most, if not all, poly(ADP-ribose) synthesis detectable in normal unperturbed cells is triggered during normal S phase by unligated Okazaki fragment intermediates of DNA replication. Our data thus identify a new role for PARP1 and suggest that unligated Okazaki fragments are a major threat to genome integrity and stability.

## Results

### PARP Activity Is Detected Primarily during S Phase at Sites of DNA Replication

**Figure 1:**
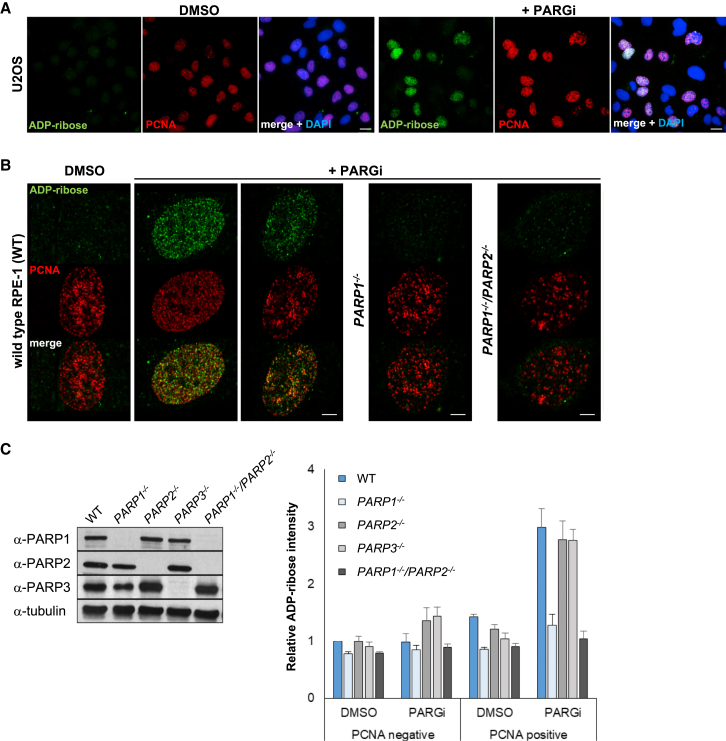
Endogenous Poly(ADP-Ribose) Is Detected Primarily during S Phase at Sites of DNA Replication (A) ADP-ribose and PCNA (indicative of S phase) immunostaining in detergent-pre-extracted U2OS cells after 30 min incubation with DMSO vehicle or PARG inhibitor (PARGi). Scale bars, 20 μm. (B) ADP-ribose and PCNA immunostaining in wild-type, *PARP1*^*−/−*^, and *PARP1*^*−/−*^*/PARP2*^*−/−*^ RPE-1 cells after 15 min incubation with DMSO vehicle or PARG inhibitor. Representative confocal images are shown. Scale bars, 5 μm. (C) Western blotting of the indicated proteins in wild-type (WT), *PARP1*^*−/−*^, *PARP2*^*−/−*^, *PARP3*^*−/−*^, and *PARP1*^*−/−*^*/PARP2*^*−/−*^ RPE-1 cell lines (left) and quantification of ADP-ribose levels in these cell lines after 15 min incubation with DMSO vehicle or PARG inhibitor in PCNA-negative (non-S phase) and PCNA-positive (S phase) cells (average of n = 4 with SEM). Representative ScanR images are shown in Figure S1B.

We reasoned that the difficulty in identifying sites of endogenous poly(ADP-ribose) synthesis in the absence of exogenous DNA damage is because it is rapidly degraded by PARG. Consequently, we attempted to detect endogenous poly(ADP-ribose) in cells following short incubation (15–60 min) with a potent PARG inhibitor (PARGi) (James et al., 2016). This approach was successful, revealing detectable levels of poly(ADP-ribose) in U2OS cells, human diploid RPE-1 cells, and a range of other cell lines (Figures 1A and S1A). Strikingly, most, if not all, of the poly(ADP-ribose) detected by this approach was present in S phase and located close to or at sites of DNA replication, as indicated by co-immunostaining with anti-proliferating cell nuclear antigen (PCNA) antibody (Figure 1A and 1B). To examine which PARP enzyme was responsible for S phase ADP-ribosylation, we employed RPE-1 cells in which PARP1, PARP2, or PARP3 was deleted (Hanzlikova et al., 2017). The S phase poly(ADP-ribose) was synthesized primarily by PARP1 because *PARP1*^*−/−*^ RPE-1 cells lacked detectable levels of S phase polymer (Figures 1B, 1C, and S1B).

### S Phase Poly(ADP-Ribose) Is Not the Result of DNA Damage or Replication Stress

**Figure 2:**
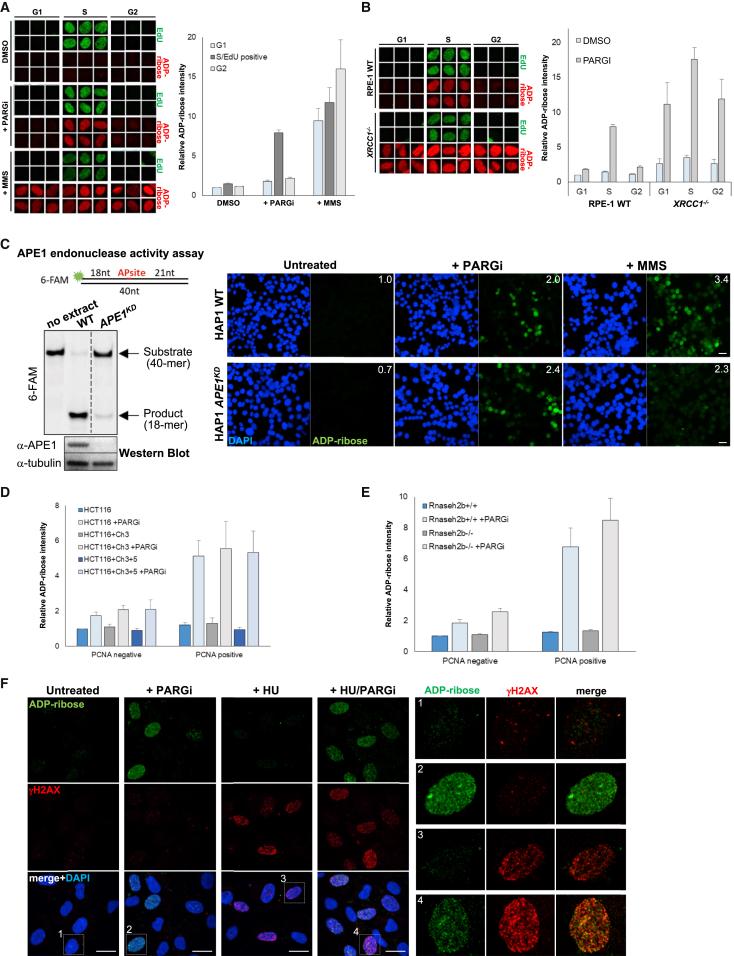
S Phase Poly(ADP-Ribose) Does Not Result from DNA Lesions or Replication Fork Stress (A) Representative ScanR images (left) and quantitation (right) of ADP-ribose in RPE-1 cells incubated for 20 min with 10 μM EdU in the absence or presence of either PARG inhibitor or MMS. Cell cycle populations were gated according to EdU positivity (S phase) and DNA content (G1 and G2) by DAPI staining (average of n = 3 with SEM). (B) Representative ScanR images and quantitation of ADP-ribose in wild-type and *XRCC1*^*−/−*^ RPE-1 cells as in (A) (average of n = 3 with SEM). (C) AP endonuclease protein (bottom left) and activity (top left) in cell extracts from wild-type and *APE1* gene-targeted human HAP1 cells additionally transfected with APE1 siRNA (denoted *APE1*^*KD*^) to further decrease APE1 levels and activity. Right: ADP-ribose immunostaining in pre-extracted untreated wild-type and *APE1*^*KD*^ cells and in cells incubated for 20 min with either PARG inhibitor or MMS. Scale bars, 20 μm. The numbers in the corners are the mean ADP-ribose intensity in all nuclei normalized to the wild-type sample, quantified in ImageJ. (D) Quantification of ADP-ribose in MMR-deficient (*MSH3* and *MLH1* mutant) HCT116 cells and their chromosome-complemented MMR-proficient counterparts HCT116+Ch3 (*MLH1*-complemented) and HCT116+Ch3+5 (*MLH1*- and *MSH3*-complemented) after 60 min incubation with or without PARG inhibitor. Cell populations were gated according to PCNA (S phase) intensity (average of n = 3 with SEM). Representative immunofluorescence images are shown in Figure S2B. (E) Quantification of ADP-ribose in PCNA-negative (non-S phase) and PCNA-positive (S phase) *Rnaseh2b*^*+/+*^ and *Rnaseh2b*^*−/−*^ mouse embryonic fibroblasts (MEFs) after incubation for 60 min with or without PARG inhibitor (average of n = 3 with SEM). Representative ScanR images are shown in Figure S2C. (F) Representative confocal images of ADP-ribose and γH2AX immunostaining in untreated RPE-1 cells and in RPE-1 cells following incubation with or without hydroxyurea (HU) for 2 hr and with or without PARG inhibitor for the final 20 min, as indicated. Scale bars, 20 μm. Insets, right: a representative and magnified cell from each image.

The appearance of ADP-ribosylation specifically in S phase was surprising because DNA damage arises stochastically throughout the cell cycle as a result of reactive endogenous electrophilic molecules and because of the intrinsic instability of DNA (Lindahl, 1993). Indeed, poly(ADP-ribose) triggered by the alkylating agent methyl methanesulfonate (MMS) was detected in G1, S, and G2 phase nuclei (Figure 2A). Additionally, cells lacking the scaffold protein XRCC1, which accelerates the repair of endogenous stochastic SSBs, exhibited elevated poly(ADP-ribose) throughout the cell cycle (Figure 2B). Together, these data suggest that the majority of detectable poly(ADP-ribose) in normal unperturbed human cells results not from stochastic DNA damage but from a source that is tightly associated with DNA replication.

To explain these results, we next considered the possibility that PARP1 was activated by one or more DNA lesions associated specifically with S phase. For example, nucleotides containing damaged or non-canonical DNA bases, such as uracil, can be incorporated during DNA replication, resulting in the elevated formation of SSBs in S phase during their excision by DNA base excision repair (BER) (Bjørås et al., 2017, Otterlei et al., 1999). However, this type of DNA base damage was not the source of S phase poly(ADP-ribose) because depletion of the APE1 endonuclease that excises abasic sites during base excision repair failed to reduce S phase poly(ADP-ribose) levels in human HAP1 cells (Figures 2C and S2A). Although these cells possess a small amount of remaining apurinic/apyrimidinic (AP) endonuclease activity (Figure 2C, left), this did not account for the persistence of S phase poly(ADP-ribose) because MMS-induced poly(ADP-ribose) was greatly reduced in the APE1-depleted cells (HAP1 *APE1*^*KD*^) (Figures 2C, right, and S2A). Another possible source of S phase poly(ADP-ribose) we considered was DNA breaks arising during the excision repair of mismatched nucleotides. However, this was not the case because *MLH1*/*MSH3* mutant DNA mismatch repair-deficient HCT116 cells (Koi et al., 1994) exhibited levels of S phase poly(ADP-ribose) similar to their repair-proficient counterparts (Figures 2D and S2B). Next we examined the possibility that S phase poly(ADP-ribose) arose during the excision repair of ribonucleotides. Ribonucleotides are misincorporated during DNA replication approximately every 5–10 kb and are excised by ribonucleotide excision repair, a process initiated by the endonuclease RNase H2 (Reijns et al., 2012, Rydberg and Game, 2002, Sparks et al., 2012). However, we failed to detect any difference between wild-type and *Rnaseh2b*^−/−^ mouse embryonic fibroblasts in levels of S phase poly(ADP-ribose), suggesting that ribonucleotide excision repair is not the source of S phase poly(ADP-ribose) (Figures 2E and S2C). This did not reflect the presence of residual RNase H2 activity because these cells lack all such activity (Reijns et al., 2012; Figure S2C, right).

We also examined the possibility that S phase ADP-ribosylation is triggered by replication stress because PARP1 has been reported to bind and be activated by stalled, reversed, and collapsed DNA replication forks (Berti et al., 2013, Bryant et al., 2009, Min et al., 2013, Ray Chaudhuri et al., 2012, Sugimura et al., 2008, Yang et al., 2004). However, the S phase poly(ADP-ribose) detected in our experiments did not result from these sources because it was not associated with γH2AX focus formation (Figure 2F), a general marker of DNA replication stress, including fork reversal (Limoli et al., 2002, Mirzoeva and Petrini, 2003, Ward and Chen, 2001, Berti et al., 2013, Ray Chaudhuri et al., 2012). In addition, treatment for 2 hr with hydroxyurea (HU) to induce DNA replication fork stress did not increase the level of S phase poly(ADP-ribose) despite increasing the level of S phase γH2AX (Figure 2F; see also Figure 4B). Collectively, these data suggest that S phase poly(ADP-ribose) arises neither from DNA lesions incorporated during DNA replication nor from DNA replication stress.

### S Phase Poly(ADP-Ribose) Is Increased by Perturbing Enzymes Involved in Okazaki Fragment Maturation

**Figure 3:**
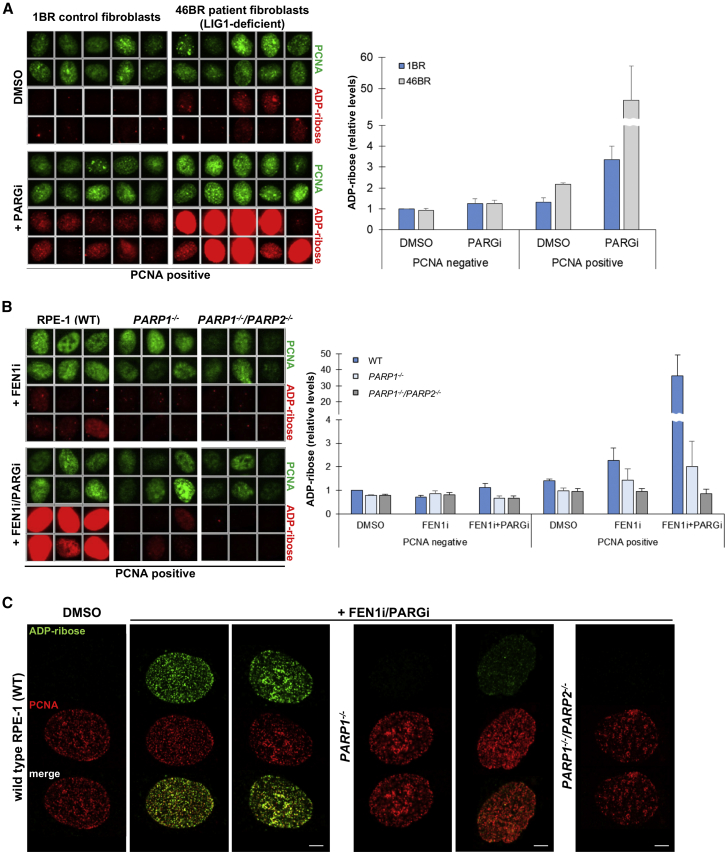
Perturbation of the DNA Replication Proteins LIG1 and FEN1 Increases S Phase Poly(ADP-Ribose) (A) Representative ScanR images (left, PCNA-positive cells only) and quantitation of ADP-ribose (right) in 1BR- and LIG1-deficient 46BR primary fibroblasts following incubation with DMSO vehicle or PARG inhibitor for 20 min. For quantitation, PCNA-negative (non-S phase) and PCNA-positive (S phase) cells were gated according to nuclear PCNA intensity. Note the break and change in scale in the y axis required to display the very high ADP-ribose level in S phase 46BR cells (average of n = 3 with SEM). (B) Representative ScanR images (left, PCNA-positive cells only) and quantification of ADP-ribose (right) as above in wild-type, *PARP1*^*−/−*^, and *PARP1*^*−/−*^*/PARP2*^*−/−*^ RPE-1 cell lines. Cells were treated with DMSO vehicle or FEN1 inhibitor (FEN1i) for 30 min, with PARG inhibitor added or not during the last 15 min, as indicated. Note the break and change in scale in the y axis required to display the very high ADP-ribose level in FEN1 inhibitor/PARG inhibitor-treated RPE-1 cells (average of n = 3 with SEM). See also Figure S5. (C) Representative confocal images of S phase (PCNA-positive) cells from the experiment in (B), illustrating the localization of poly(ADP-ribose) with PCNA at DNA replication sites. Scale bars, 5 μm.

Next we considered the possibility that S phase poly(ADP-ribose) synthesis occurs during DNA replication at one or more canonical DNA replication intermediates. To test this, we first employed 46BR human fibroblasts from a patient harboring mutated DNA ligase I (LIG1), the enzyme that ligates Okazaki fragments during DNA replication (Barnes et al., 1992, Henderson et al., 1985, Levin et al., 1997, Levin et al., 2000, Lönn et al., 1989, Prigent et al., 1994). Strikingly, 46BR cells exhibited S phase poly(ADP-ribose) levels that were ∼14-fold higher than in normal human fibroblasts (1BR) when poly(ADP-ribose) degradation was prevented by PARG inhibition (Figure 3A). Moreover, elevated S phase poly(ADP-ribose) was detectable in 46BR cells even in the absence of PARG inhibition. These results were not restricted to LIG1 mutation because similar results were observed when we depleted LIG1 using small interfering RNAs (siRNAs) (Figure S3A). Importantly, we did not detect an increase in poly(ADP-ribose) levels in LIG1-defective cells that did not stain positive for PCNA or 5-ethynyl-2’-deoxyuridine (EdU) (Figures 3A and S3A), indicating that the increased poly(ADP-ribose) was S phase-specific and consistent with it resulting from the increased unligated Okazaki fragments that are present in LIG1-defective cells (Barnes et al., 1992, Henderson et al., 1985, Levin et al., 2000, Levin et al., 1997, Lönn et al., 1989, Prigent et al., 1994). To test this possibility further, we examined poly(ADP-ribose) levels in RPE-1 cells in which we transiently inhibited FEN1, the nuclease that excises 5′ flaps during Okazaki fragment processing prior to their ligation by LIG1 (Goulian et al., 1990, Harrington and Lieber, 1994, Ishimi et al., 1988, Robins et al., 1994, Waga et al., 1994). Indeed, similar to LIG1 perturbation, incubation with FEN1 inhibitor (FEN1i) (Exell et al., 2016) in the presence of PARG inhibitor increased the level of S phase poly(ADP-ribose) ∼12-fold relative to the level of S phase poly(ADP-ribose) detected in the presence of PARG inhibitor alone (Figures 3B and S5). Again, the elevated poly(ADP-ribose) was observed only in S phase and localized extensively with PCNA (Figures 3B and 3C). The S phase poly(ADP-ribose) induced by the FEN1 inhibitor was primarily dependent on PARP1, although complete ablation in some cells required the additional deletion of PARP2 (Figures 3B and 3C). The effect of the FEN1 inhibitor on levels of S phase poly(ADP-ribose) was not a non-specific reflection of increased DNA replication stress because, similar to the PARG inhibitor, there was no obvious effect of the inhibitor on either DNA replication rate or on γH2AX focus formation under the conditions employed (Figures S3B and S3C). Rather, these data implicate S phase poly(ADP-ribose) as a molecular indicator of unligated Okazaki fragments.

### PARP Activity during S Phase Is Prevented by Suppressing Okazaki Fragment Formation

**Figure 4:**
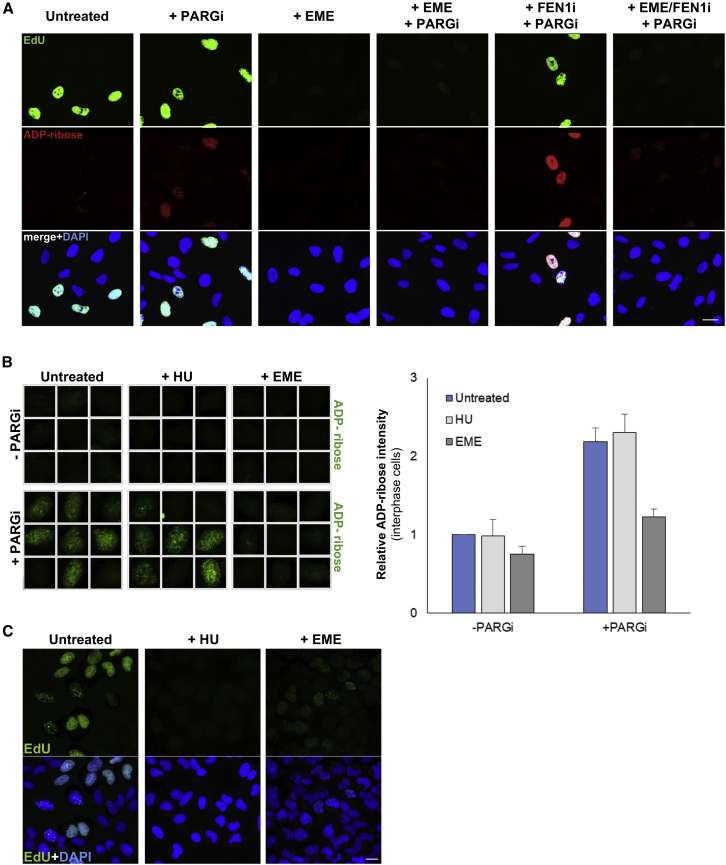
Suppression of Okazaki Fragment Formation with Emetine Prevents S Phase ADP-Ribosylation (A) Indirect immunofluorescence imaging of ADP-ribose and EdU in wild-type RPE-1 cells incubated or not with emetine (EME) and/or FEN1 inhibitor for 45 min as indicated, with or without PARG inhibitor added during the final 20 min. EdU was added to all samples during the last 20 min to detect DNA synthesis. Scale bar, 20 μm. (B) Representative ScanR images (left) and quantification (right) of mean ADP-ribose levels in pre-extracted U2OS interphase cells treated or not for 2 hr with hydroxyurea or for 1 hr with emetine, as indicated (average of n = 3 with SEM). PARG inhibitor was added or not, as indicated, during the final 30 min. Note that the ADP-ribose quantifications are the mean levels across all interphase cells (not just S phase cells). (C) EdU labeling in U2OS cells treated or not with hydroxyurea for 2 hr or emetine for 1 hr. Cells were incubated with 10 μM EdU for the final 20 min. Scale bar, 20 μm.

To confirm that Okazaki fragments were the source of S phase poly(ADP-ribose), we employed the DNA replication inhibitor emetine. Emetine is an inhibitor of DNA replication that prevents the formation of Okazaki fragments, uncoupling leading and lagging strand DNA replication (Burhans et al., 1991). The ability of this drug to inhibit lagging strand synthesis has been exploited previously to map eukaryotic replication origins because it enables the selective pulse-labeling of nascent leading strands that can be used as sequence-specific probes (Aladjem et al., 1998, Aladjem and Wahl, 1997, Burhans et al., 1991, Handeli et al., 1989, Kitsberg et al., 1993). Strikingly, short incubation with emetine (EME) completely blocked the appearance of S phase poly(ADP-ribose) in both RPE-1 cells (Figure 4A) and U2OS cells (Figures 4B). Moreover, emetine ablated most of the S phase poly(ADP-ribose) triggered by the FEN1 inhibitor, confirming that this drug prevented PARP activation at sites of unligated Okazaki fragments (Figure 4A). This result did not reflect a non-specific effect of emetine on PARP activity because emetine did not block poly(ADP-ribose) synthesis at sites of stochastic DNA damage induced by MMS (Figure S4). Nor did this result reflect a non-specific effect of emetine on DNA replication because the DNA replication inhibitor hydroxyurea did not reduce the level of S phase poly(ADP-ribose) despite inhibiting DNA synthesis to a greater extent than emetine, as measured by EdU incorporation (Figure 4C).

### S Phase Poly(ADP-Ribose) Results in Recruitment of the SSBR Scaffold Protein XRCC1

**Figure 5:**
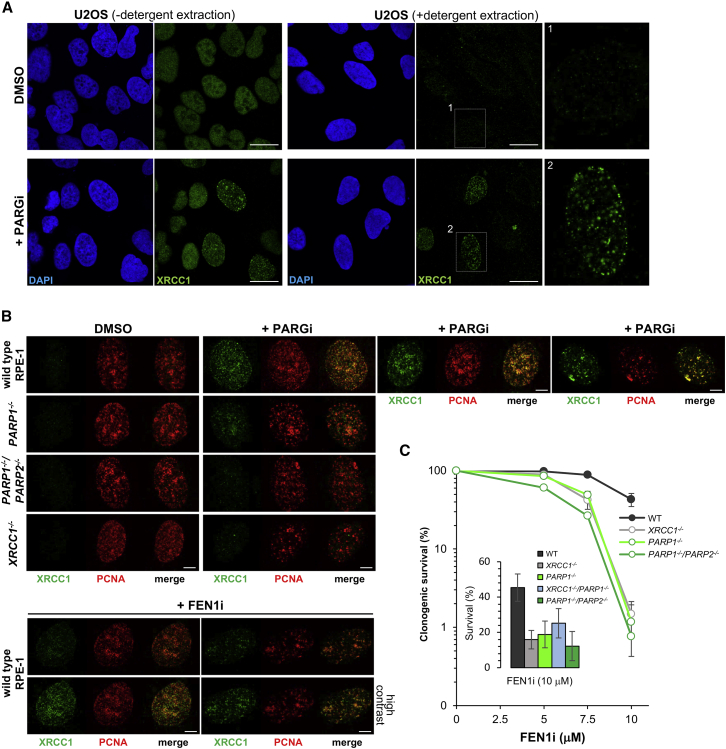
S Phase PARP Activity Recruits the DNA Repair Protein XRCC1 and Protects Cells from the Effect of the FEN1 Inhibitor (A) Representative confocal images of XRCC1 immunostaining in U2OS cells after incubation for 30 min in the presence of DMSO vehicle or PARG inhibitor. Cells were fixed immediately to detect total XRCC1 or were pre-extracted with Triton X-100 before fixation to detect chromatin-bound XRCC1. Magnified images of two representative cells are shown (right). Scale bars, 20 μm. (B) Representative confocal images of XRCC1 and PCNA immunostaining in PCNA-positive (S phase) wild-type, *PARP1*^*−/−*^, *PARP1*^*−/−*^*/PARP2*^*−/−*^, and *XRCC1*^*−/−*^ RPE-1 cells after incubation in the presence or absence of PARG inhibitor for 15 min (top), as indicated, or in wild-type RPE-1 cells following incubation in the presence of FEN1 inhibitor for 30 min (bottom). Scale bars, 5 μm. (C) Clonogenic survival of wild-type, *PARP1*^*−/−*^, *PARP1*^*−/−*^*/PARP2*^*−/−*^, and *XRCC1*^*−/−*^ RPE-1 cells following incubation in the presence of the indicated concentrations of FEN1 inhibitor. Data are the mean ± SEM of three independent experiments. The inset shows an independent set (n = 3) of experiments in which the above cell lines and, additionally, *XRCC1*^*−/−*^*/PARP1*^*−/−*^ cells were incubated with 10 μM FEN1 inhibitor.

The data described above implicate PARP1 and PARP2 as sensors of incompletely processed Okazaki fragments. Why are these enzymes activated during unperturbed S phase in which the canonical pathway for processing Okazaki fragments is present? Because replication of the human genome requires the synthesis and ligation of 30–50 million Okazaki fragments, we considered the possibility that a sub-fraction of these fragments escapes canonical processing by FEN1 and/or LIG1. We posited that S phase poly(ADP-ribose) might signal the presence of these incomplete DNA replication intermediates and facilitate recruitment of the SSBR machinery to complete their ligation. In agreement with this idea, we detected the presence of the SSBR scaffold protein XRCC1 in foci that were resistant to detergent extraction in a sub-population of U2OS cells, consistent with its presence in DNA replication foci, when poly(ADP-ribose) degradation was prevented (Figure 5A). Similar results were observed in RPE-1 cells, which we additionally co-stained with PCNA to identify sites of DNA replication (Figure 5B). The presence of XRCC1 in DNA replication foci was reduced by deletion of PARP1 and ablated by additional deletion of PARP2 (Figure 5B), consistent with the overlapping role of these two enzymes in facilitating XRCC1 recruitment (Hanzlikova et al., 2017). Notably, XRCC1 recruitment was detected even in the absence of PARG inhibitor when FEN1 was inhibited to increase the level of unligated Okazaki fragments (Figure 5B, bottom).

### Loss of PARP1, PARP2, and/or XRCC1 Results in Hypersensitivity to the FEN1 Inhibitor

Finally, to examine the importance of XRCC1-dependent SSBR for cellular tolerance to unprocessed Okazaki fragments, we compared wild-type RPE-1 cells and cells lacking PARP1, PARP2, and/or XRCC1 for hypersensitivity to FEN1 inhibition. Indeed, *XRCC1*^*−/−*^, *PARP1*^*−/−*^, and *PARP1*^*−/−*^*/PARP2*^*−/−*^ RPE-1 cells were each more sensitive to FEN1 inhibitor than wild-type RPE-1 cells (Figure 5C). Notably, *XRCC1*^*−/−*^*/PARP1*^*−/−*^ RPE-1 cells in which both proteins were absent were no more sensitive than either single mutant cell line, confirming that these proteins function in the same pathway (Figure 5C, inset). Importantly, the hypersensitivity of *PARP1*^*−/−*^ and *XRCC1*^*−/−*^ cells did not reflect a role for PARP1 and XRCC1 in the repair of stochastic DNA damage induced by the FEN1 inhibitor. This is because the elevated poly(ADP-ribose) signal in *XRCC1*^*−/−*^ cells outside of S phase, which is indicative of stochastic DNA damage, was not further increased by the FEN1 inhibitor (Figure S5).

In summary, we show here that extensive poly(ADP-ribose) synthesis is a feature of normal unperturbed S phase and signals the presence of unligated Okazaki fragments. We propose that the synthesis of S phase poly(ADP-ribose) results in the recruitment of PARP-dependent SSBR, which, we conclude, is a non-canonical pathway for Okazaki fragment maturation.

## Discussion

The use of a highly selective PARG inhibitor in this work has uncovered high levels of poly(ADP-ribose) during normal S phase at sites of DNA replication. This observation was applicable to a range of different cell lines, suggesting that the synthesis of poly(ADP-ribose) is a common feature of normal S phase. The absence of detectable poly(ADP-ribose) in cells that have not been incubated with the PARG inhibitor likely reflects the high catalytic activity of this enzyme and possibly also its recruitment into the replisome by interaction with PCNA (Kaufmann et al., 2017, Mortusewicz et al., 2011). Our data are consistent with a previous report in which poly(ADP-ribose) was detected in S phase cells by suppressing PARG activity with siRNA (Ray Chaudhuri et al., 2015). However, the latter study employed conditions under which PARG activity was suppressed for ∼24 hr, which itself induces DNA replication fork damage (Gravells et al., 2017, Illuzzi et al., 2014, Ray Chaudhuri et al., 2015). In contrast, the short-term (15–60 min) suppression of PARG activity employed in our experiments does not induce replication fork damage (James et al., 2016), an observation confirmed in our experiments by the absence of γH2AX induction or any major effect on DNA replication rate. We thus conclude that the S phase poly(ADP-ribose) detected in this work reflects genuine sites of PARP1 activity during normal S phase rather than additional sites induced by the PARG inhibitor.

Interestingly, in SSBR-defective *XRCC1*^*−/−*^ cells, incubation with the PARG inhibitor additionally uncovered the presence of poly(ADP-ribose) outside of S phase at stochastic SSBs in G1 and G2. However, this poly(ADP-ribose) is different from that observed in S phase because it was not detected in SSBR-proficient wild-type cells. This suggests that the source of PARP activity in S phase is not stochastic DNA damage but, rather, is a DNA structure that arises specifically during DNA replication. Although there are several types of S phase-specific DNA lesions that could trigger poly(ADP-ribose) synthesis during their excision repair, we were unable to alter S phase poly(ADP-ribose) by depleting or deleting the enzymes required for their removal. Similarly, although PARP1 and/or PARP2 are also activated at stalled or damaged replication forks, the S phase poly(ADP-ribose) detected here was not triggered by such structures because it was not associated with γH2AX and because the deliberate induction of replication fork stress by short-term incubation with hydroxyurea did not induce additional S phase poly(ADP-ribose). In contrast, however, perturbation of the Okazaki fragment processing enzymes FEN1 or LIG1 triggered large increases in S phase poly(ADP-ribose) levels, strongly implicating unligated Okazaki fragments as a potent source of S phase poly(ADP-ribose) synthesis. Although FEN1 and LIG1 are also implicated in long-patch DNA base excision repair (Klungland and Lindahl, 1997, Levin et al., 2000, Prasad et al., 2000) this role cannot account for their effect on S phase poly(ADP-ribose). This is because the perturbation of FEN1 and LIG1 in our experiments increased poly(ADP-ribose) levels only during S phase, whereas long-patch base excision repair is also operative outside of S phase (Akbari et al., 2009, Kleppa et al., 2012, Woodrick et al., 2017). In addition, long-patch base excision repair is largely dependent on AP endonuclease activity, which, we demonstrated, does not influence the level of S phase poly(ADP-ribose). Nevertheless, to confirm that unligated Okazaki fragments were the source of S phase poly(ADP-ribose), we employed emetine, an inhibitor of DNA replication that, when employed for short periods, selectively inhibits the synthesis of Okazaki fragments (Burhans et al., 1991). Similar to hydroxyurea, emetine uncouples leading strand and lagging strand replication and greatly reduces the overall rate of DNA synthesis. Critically, however, although the residual nascent DNA in hydroxyurea-treated cells is enriched for short Okazaki-like DNA fragments (Laipis and Levine, 1973, Magnusson, 1973a, Magnusson, 1973b, Martin et al., 1977, Radford et al., 1982), the residual nascent DNA in emetine-treated cells results only from leading strand replication (Burhans et al., 1991). Consistent with this, emetine almost completely prevented the appearance of poly(ADP-ribose) in S phase, even in cells in which polymer levels were elevated by incubation with the FEN1 inhibitor. This contrasted markedly with hydroxyurea, which, despite reducing total DNA synthesis to a similar level as emetine, did not alter the level of S phase poly(ADP-ribose).

Why do cells require PARP1 to detect unligated Okazaki fragments? Although the canonical pathway for lagging DNA replication is highly coordinated, it is possible that this pathway is unable to process all of the 30–50 million Okazaki fragments that arise during each human S phase and, thus, that other mechanisms are required to detect and repair these structures. Consistent with this idea, it has been estimated that 15%–30% of human DNA polymerase δ molecules dissociate before encountering a downstream Okazaki fragment (Hedglin et al., 2016). In addition, single-strand gaps in nascent DNA can arise by replicative bypass of lesions or other obstructions in DNA template strands (Langston and O’Donnell, 2006, Marians, 2018), suggesting that S phase poly(ADP-ribose) synthesis might be triggered by gaps in either the leading or lagging nascent strands. Our finding that XRCC1 was recruited at sites of DNA replication by stimulating poly(ADP-ribose) synthesis with the FEN1 inhibitor is consistent with a role for PARP-dependent SSBR in processing unligated Okazaki fragments, as is our observation that deletion of PARP1, PARP2, and/or XRCC1 results in hypersensitivity to this inhibitor. Similarly, this idea is consistent with the established sensitivity of *LIG1*-mutated 46BR cells to the PARP inhibitor (Lehmann et al., 1988, Teo et al., 1983) and with the dependence of chicken DT40 cells lacking LIG1 on the XRCC1 protein partner LIG3 for viability (Arakawa and Iliakis, 2015). Finally, it is worth noting that treatment of human cells with the PARP inhibitor 3-aminobenzamide was reported more than 30 years ago to result in the accumulation of 10-kb nascent DNA fragments, consistent with a requirement of PARP1 activity for the maturation of a subset of nascent DNA replication intermediates (Lönn and Lönn, 1985).

Finally, our data have important implications concerning the impact of unligated Okazaki fragments. For example, it has been suggested that the lethality invoked by complete loss of XRCC1 or PARP activity in mouse embryos undergoing rapid cell divisions during gastrulation could reflect unrepaired stochastic SSBs that impede DNA replication (Ménissier de Murcia et al., 2003, Tebbs et al., 1999). Although this argument is attractive, our data suggest that, in addition to stochastic SSBs, unligated Okazaki fragments are a likely contributing factor to the lethality observed in embryos lacking PARP-dependent SSBR. Similarly, a role for PARP activity in the repair of Okazaki fragments may have relevance to the established hypersensitivity of HR-defective cancer cells to PARP inhibition (Bryant et al., 2005, Farmer et al., 2005). Although PARP inhibitors are now exploited clinically to treat *BRCA1*- and *BRCA2*-mutated cancers, the nature of the DNA structures on which PARP enzymes are “trapped” by these inhibitors is unclear. Our data implicate unligated Okazaki fragments as one such structure. We suggest that unligated Okazaki fragments that are trapped by the PARP inhibitor require HR-mediated repair for their removal, either directly as single-strand gaps or following their conversion into DSBs by nucleases or DNA replication fork collapse, as has been demonstrated in *E. coli* (Kouzminova and Kuzminov, 2012, Kuzminov, 2001). That unligated Okazaki fragments can also trigger HR-mediated repair in human cells is consistent with the observation that *LIG1*-mutated 46BR cells exhibit elevated levels of baseline sister chromatid exchange (Henderson et al., 1985) and with the observation that the FEN1 inhibitor induces RAD51 focus formation or cell death in *BRCA2*-proficient and -deficient cells, respectively (Ward et al., 2017).

In summary, we show here that poly(ADP-ribose) is detected primarily at sites of DNA replication in normal human S phase, and we implicate PARP-dependent SSBR machinery as a novel “backup” pathway for processing unligated Okazaki fragments.

## STAR★Methods

### Key Resources Table

**Table:** 

REAGENT or RESOURCE	SOURCE	IDENTIFIER
**Antibodies**		

Rabbit polyclonal anti-XRCC1	Millipore	Cat# ABC738
Mouse monoclonal anti-PARP1	Serotec	Cat# MCA1522G
Rabbit polyclonal anti-PARP2	Active Motif	Cat# 39743
Rabbit polyclonal anti-PARP3	a gift from F. Dantzer	4699
Rabbit polyclonal anti-poly(ADP-ribose)	Trevigen	Cat# 4336
Rabbit Fc-fused anti-pan-ADP-ribose binding reagent	Millipore	Cat# MABE1016
Rabbit polyclonal anti-APE1	Invitrogen	Cat# PA517233
Rabbit polyclonal anti-mouse RNase H2 complex	a gift from A. Jackson, Reijns et al., 2012	62
Mouse monoclonal anti-γH2AX	Millipore	Cat# 05-636
Mouse monoclonal anti-PCNA	Santa Cruz	Cat# sc-56
Rat polyclonal anti-α-tubulin	Abcam	Cat# ab6160
HRP-conjugated goat anti-rabbit	Bio-Rad	Cat# 170-6515
HRP-conjugated goat anti-mouse	Bio-Rad	Cat# 170-6516
HRP-conjugated rabbit anti-rat	Abcam	Cat# ab6734

**Biological Samples**		

1BR human fibroblasts	*GDSC cell bank*	
46BR human fibroblasts	Henderson et al., 1985	

**Chemicals, Peptides, and Recombinant Proteins**		

PARG inhibitor	Tocris	PDD 0017273; 5952
PARG inhibitor	a gift from D. James, James et al., 2016	PDD 00017272
FEN1 inhibitor	This paper	Exell et al., 2016
Methyl methansulfonate (MMS)	Sigma-Aldrich	Cat# 129925
Hydroxyurea	Sigma-Aldrich	Cat# H8627
Emetine	Sigma-Aldrich	Cat# E2375
Camptothecin (CPT)	Sigma-Aldrich	Cat# C9911

**Critical Commercial Assays**		

Click-iT EdU Alexa Fluor 488 Imaging Kit	Invitrogen	Cat# C10337

**Deposited Data**		

Original imaging data	This paper	

**Experimental Models: Cell Lines**		

Human: hTERT RPE-1	ATCC	CRL-4000
Human: *PARP1*^*−/−*^	Hanzlikova et al., 2017	
Human: *PARP2*^*−/−*^	Hanzlikova et al., 2017	
Human: *PARP3*^*−/−*^	Hanzlikova et al., 2017	
Human: *PARP1*^*−/−*^/*PARP2*^*−/−*^	Hanzlikova et al., 2017	
Human: *XRCC1*^*−/−*^	Hoch et al., 2017	
Human: *XRCC1*^*−/−*^/*PARP1*^*−/−*^	Hoch et al., 2017	
Human: U2OS	ATCC	HTB-96
Human: HeLa	ATCC	CCL-2
Mouse: *Rnaseh2b*^*+/+*^	Reijns et al., 2012	
Mouse: *Rnaseh2b*^*−/−*^	Reijns et al., 2012	
Human: HAP1 parental control	Horizon	C631
Human: *APE1* knockout cell line 2bp deletion	Horizon	HZGHC005289c003
Human: HCT116	Koi et al., 1994	
Human: HCT116+Ch3	Koi et al., 1994	
Human: HCT116+Ch3+5	Koi et al., 1994	

**Oligonucleotides**		

si*NT* (non-targeting siRNA)	Dharmacon	ON-TARGETplus
si*APE1*	Dharmacon	SMARTpool
si*LIG1*	Dharmacon	SMARTpool
si*LIG1* (#1): GGCAUGAUCCUGAAGCAGA	Dharmacon	N/A
si*PARP1*	Dharmacon	SMARTpool
RiboG oligo: 5′-(6-FAM)-TAGCATCGATCAGTCCTC(rG)GAGG TCTAGCATCGTTAGTCA-(TAMRA)-3′	Midland Certified Reagent Company	N/A
AP oligo: 5′-(6-FAM)-TAGCATCGATCAGTCCTC(APsite)GAG GTCTAGCATCGTTAGTCA-(TAMRA)-3′	Midland Certified Reagent Company	N/A
Complementary oligo: 5′-TGACTAACGATGCTAGACCTCTGA GGACTGATCGATGCTA-3′	Midland Certified Reagent Company	N/A
Competitor oligo: 5′-AAAGATCACAAGCATAAAGAGACAGG-3′	Midland Certified Reagent Company	N/A

**Software and Algorithms**		

ScanR Analysis Software	Olympus	
ImageJ	NIH	
PharosFX Molecular Imager System	Bio-Rad	

### Contact for Reagent and Resource Sharing

Further information and requests for resources and reagents should be directed to Keith W. Caldecott (k.w.caldecott@sussex.ac.uk).

### Experimental Model and Subject Details

#### Chemicals

PARG inhibitor was purchased from Tocris (PDD 0017273; 5952) or was as a gift from Dominic James (PDD 00017272) (similar data were obtained with both). FEN1 inhibitor was synthesized as described previously [compound 24 in (Tumey et al., 2005)]. Both inhibitors were dissolved in dimethyl sulfoxide (DMSO) to a working concentration of 10 mM. Methyl methansulfonate (MMS) was dissolved directly into culture medium, 2 M hydroxyurea (HU) solution was prepared in water and 2 mM emetine in PBS. 10 mM camptothecin stock was in DMSO. *Final concentrations were as follows:* 10 μM PARG inhibitor, 10 μM FEN1 inhibitor, 0.2 mg/ml MMS, 2 mM hydroxyurea, 2 μM emetine and 10 μM CPT.

#### Cell culture

Human wild-type, *PARP1*^*−/−*^, *PARP2*^*−/−*^, *PARP3*^*−/−*^, *PARP1*^*−/−*^/*PARP2*^*−/−*^, *XRCC1*^*−/−*^ and *XRCC1*^*−/−*^/*PARP1*^*−/−*^ hTERT RPE-1 cell lines have been described previously (Hanzlikova et al., 2017, Hoch et al., 2017). Cells were cultured in Dulbecco’s Modified Eagle’s Medium (DMEM/F12) supplemented with 10% fetal calf serum (FCS) and 0.01 mg/ml hygromycin. Human U2OS cells, HeLa and mouse embryonic fibroblasts (MEFs) from wild-type or *Rnaseh2b*^*−/−*^ mice were grown in DMEM containing 10% FCS, 2 mM L-glutamine, and the antibiotics *penicillin* (100 U/ml) and *streptomycin* (100 μg/ml) (*Pen/Strep*). Wild-type and *APE1* gene-targeted (2 bp deletion) human HAP1 cells were cultured in Iscove’s Modified Dulbecco’s Medium (IMDM) with 10% FCS and the antibiotics *Pen/Strep*. *MLH1*/*MSH3*-deficient HCT116 cells harboring *MLH1* and *MSH3* mutations on chromosome 3 and 5, respectively, and derivatives in which wild-type *MLH1* (HCT116+Ch3) or both *MLH1* and *MSH3* (HCT116+Ch3+5) were introduced by chromosome transfer (Koi et al., 1994) were grown in McCoy’s 5a with L-glutamine, 10% FCS and *Pen/Strep*. Primary human fibroblasts, 1BR and (LIG1)-deficient 46BR cells were cultured in Minimum Essential Media (MEM) supplemented with 15% FCS, 2 mM L-glutamine, and the antibiotics *Pen/Strep*.

### Method Details

#### siRNA and transfection

Non-targeting siRNA (ON-TARGETplus) and SMARTpool siRNA against APE1, LIG1, or PARP1, or the single LIG1 siRNA (#1) were reverse-transfected into the cells using Lipofectamine^®^ RNAiMAX (Invitrogen) according to the manufacturer’s instructions. All experiments were carried out 72 hr post-transfection.

#### APE1 and RNase H2 *in vitro* assays

The substrate for the *in vitro* RNase H2 assay was prepared by annealing equimolar amounts of RiboG oligo and Complementary oligo (Midland Certified Reagent Company) in 10 mM Tris pH 8.0, 200 mM NaCl, 1 mM EDTA. The substrate for the *in vitro* APE1 assay was prepared by annealing equimolar AP oligo and Complementary oligo in 10 mM Tris pH 7.5, 200 mM NaCl, 1 mM EDTA. Oligonucleotides were incubated at 95°C for 3 min and allowed to slowly cool to RT. Cells were trypsinised, washed in PBS, resuspended in lysis buffer [25 mM Tris, pH 7.5, 10 mM EDTA, 10 mM EGTA, 100 mM NaCl, 1% Triton X-100, cOmplete protease inhibitors (Roche)], and incubated on ice for 15 min. Lysates were centrifuged at 16.800 g for 20 min at 4°C. 50 nM substrates were incubated with 0.5, 1 or 2 μg of cell extract in RNase H2 reaction buffer (50 mM Tris, pH 7.5, 60 mM KCl, 10 mM MgCl_2_, 0.01% BSA, 0.01% Triton X-100, 1 μM Competitor oligo) or 1 μg of cell extract in APE1 reaction buffer (20 mM Tris, pH 7.5, 100 mM KCl, 10 mM MgCl_2_, 0.5 mM DTT, 0.25% polyvinyl alcohol, 1 μM Competitor oligo) to measure RNase H2 or APE1 activity, respectively. 50 μL reactions were incubated at 37°C for 60 min for RNase H2 assay or 30 min for APE1 assay. Reactions were terminated by addition of 50 μL of quenching buffer (90% formamide, 35 mM EDTA, 300 mM NaOH, 0.006% Orange G). 10 μL of each reaction was loaded on 20% denaturing polyacrylamide gel and analyzed by PharosFX Molecular Imager System (Bio-Rad).

#### SDS-PAGE and western blotting

Cells were collected and lysed in SDS sample buffer (2% SDS, 10% glycerol, 50 mM Tric-Cl, pH 6.8), denatured for 10 min at 95°C, and sonicated for 30 s using Bioruptor^®^ Pico (Diagenode). Protein concentrations were determined using the BCA assay (Pierce). DTT and bromophenol blue were added to samples which were subjected to SDS-PAGE, proteins transferred onto nitrocellulose membrane and detected by relevant specific antibodies combined with horseradish peroxidase-conjugated secondary antibodies. Peroxidase activity was detected by ECL reagent (GE Healthcare) and Amersham Hyperfilm ECL (GE Healthcare).

#### Immunofluorescence and microscopy

Cells cultured on glass coverslips were fixed with 4% formaldehyde in PBS for 10 min at room temperature (RT) and subsequently permeabilized by a 5 min incubation in ice-cold methanol/acetone solution (1:1). Where required (mainly in experiments involving PCNA labeling), before fixation, cells were pre-extracted for 2 min on ice in 0.2% Triton X-100. After blocking the cells with 10% fetal calf serum, slides were incubated with the primary antibody (60 min, RT), followed by washing (3 × 5 min in PBS) and then incubation with the appropriate fluorescently labeled secondary antibody (60 min, RT). Coverslips were washed (3 × 5 min in PBS), stained with DAPI (1 μg/ml in water, 2 min) and mounted using VECTASHIELD (Vector Laboratories). EdU labeling was performed using Click-iT EdU Alexa Fluor 488 Imaging Kit according to the manufacturer’s instructions. High-resolution pictures were acquired by imaging with a Leica SP8 confocal microscope or a Leica DM6000 fluorescence microscope. Automated wide-field image acquisition was done using Olympus ScanR high-content screening station equipped with a motorized stage and 40x objective. Nuclei were identified based on the DAPI signal and EdU or PCNA positive cells were gated and quantified using ScanR Analysis Software. At least 1,000 nuclei for interphase cells were counted per condition in three or four independent experiments. Data are represented as mean ± SEM.

#### Clonogenic survival assays

Clonogenic survival was determined by colony formation assays. Wild-type human hTERT RPE-1 cells and gene-edited derivatives were plated in 10 mm dishes and 4 hr later treated with indicated concentrations of FEN1 inhibitor. Cells were incubated with drug-containing media for 10-14 days and then fixed in 100% ethanol and stained with 0.05% crystal violet solution. The surviving fraction at each dose was calculated by dividing the average number of colonies in treated dishes by the average number in untreated dishes.

## Supplemental Information

Document S1. Figures S1–S5

Document S2. Article plus Supplemental Information
