# Supplementary material for: The Importance of Poly(ADP-Ribose) Polymerase as a Sensor of Unligated Okazaki Fragments during DNA Replication
Source: Mol Cell. 2018 Jul 19;71(2):319–331.e3. doi: 10.1016/j.molcel.2018.06.004 (PMC6060609; doi:10.1016/j.molcel.2018.06.004)
Supplement: Document S1. Figures S1–S5 [file mmc1.pdf]

Molecular Cell, Volume *71*

Supplemental Information

The Importance of Poly(ADP-Ribose) Polymerase as a Sensor of Unligated Okazaki Fragments during DNA Replication

Hana Hanzlikova, Ilona Kalasova, Annie A. Demin, Lewis E. Pennicott, Zuzana Cihlarova, and Keith W. Caldecott

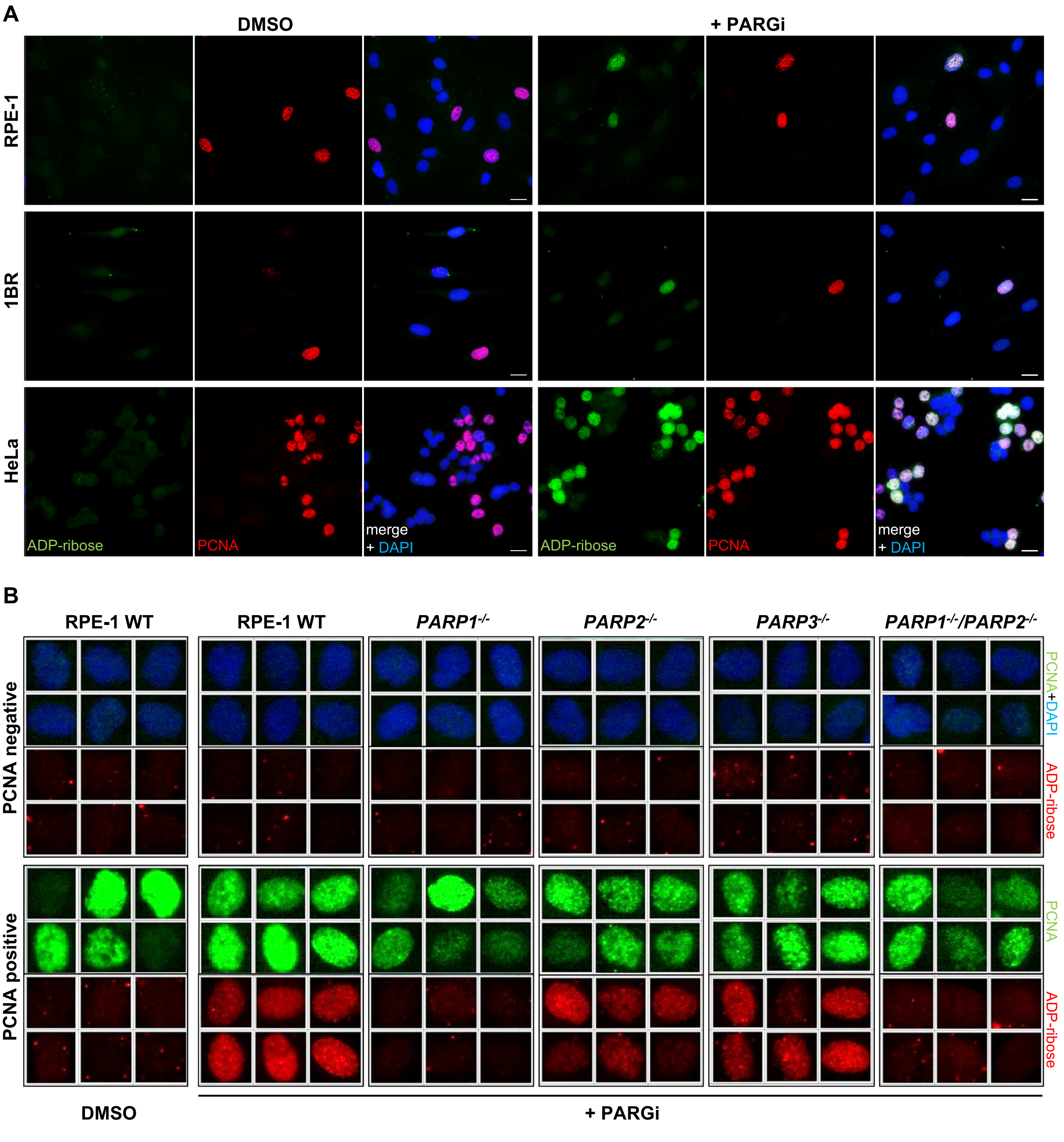

Figure S1. S phase poly(ADP-ribose) is present in multiple cell types and is synthesized primarily by PARP1 (related to Figure 1).

(A) Indirect immunofluorescence imaging of ADP-ribose and PCNA (to identify S phase cells) in RPE-1, 1BR and HeLa cells after incubation for 30 min with DMSO vehicle or PARGi. Scale bar, 20 μm.

(B) Representative ScanR images from the experiment in Figure 1C. ADP-ribose and PCNA levels in wild type (WT), *PARP1*$^{-/-}$, *PARP2*$^{-/-}$, *PARP3*$^{-/-}$ and *PARP1*$^{-/-}$/*PARP2*$^{-/-}$ RPE-1 clonal cell lines following incubation for 15 min with DMSO vehicle or with PARGi.

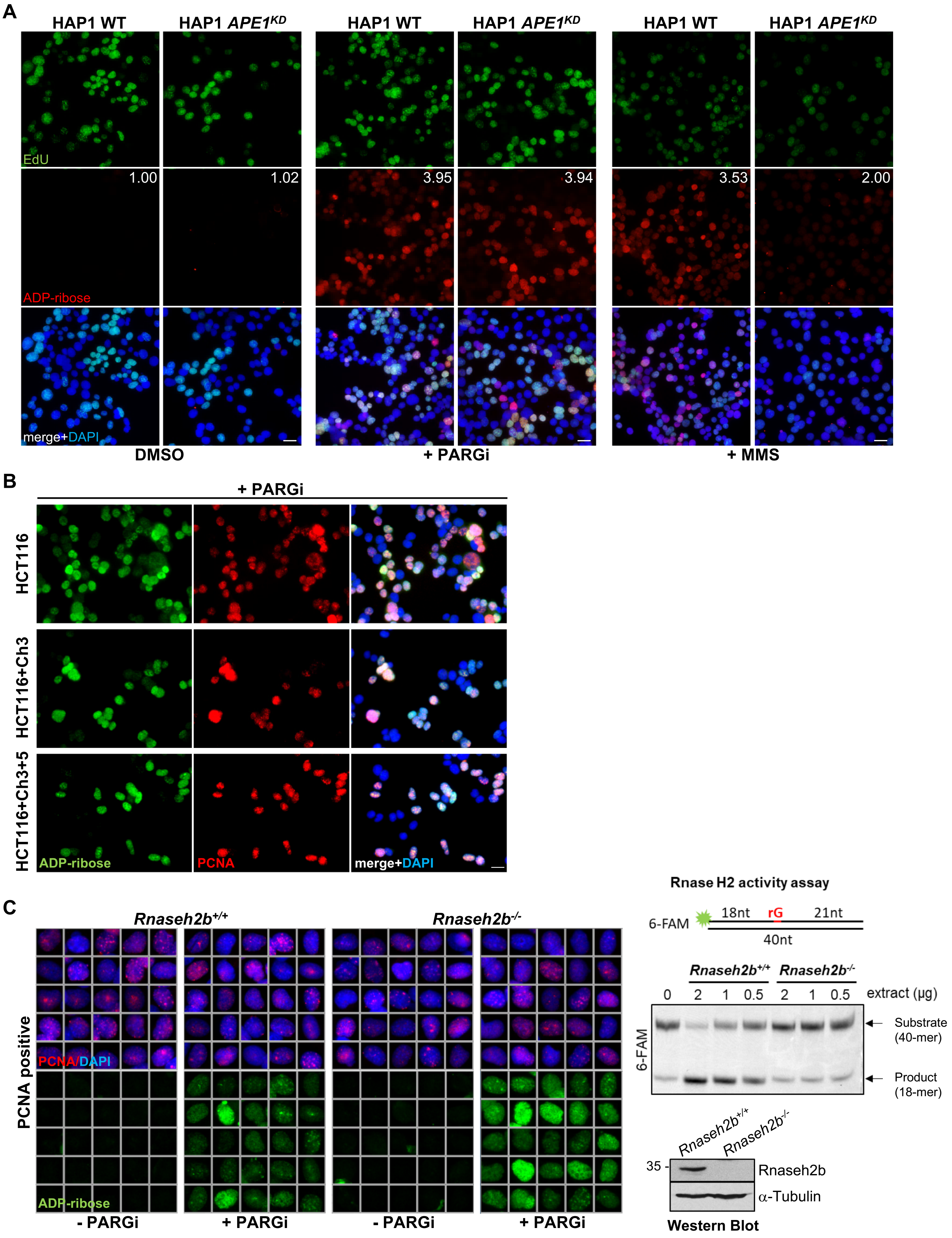
A
HAP1 WT
HAP1 $APE1^{KD}$
EdU
ADP-ribose
merge+DAPI
1.00
1.02
3.95
3.94
3.53
2.00
DMSO
+ PARGi
+ MMS
B
+ PARGi
HCT116
HCT116+Ch3
HCT116+Ch3+5
ADP-ribose
PCNA
merge+DAPI
C
$Rnaseh2b^{+/+}$
$Rnaseh2b^{-/-}$
PCNA positive
PCNA/DAPI
ADP-ribose
- PARGi
+ PARGi
Rnase H2 activity assay
6-FAM
18nt
rG
21nt
40nt
extract (μg)
0
2
1
0.5
6-FAM
Substrate (40-mer)
Product (18-mer)
35
Rnaseh2b
α-Tubulin
Western Blot

Figure S2. ADP-ribosylation in base excision, DNA mismatch and ribonucleotide excision repair-defective cell lines (related to Figure 2).

(A) Indirect immunofluorescence imaging of ADP-ribose and EdU in wild type (WT) human HAP1 cells, and in *APE1* gene-targeted HAP1 cells additionally transfected with APE1 siRNA ($APE1^{KD}$). Cells were incubated for 20 min with 10 μM EdU in the absence or presence of either PARGi or MMS, as indicated. Scale bar, 20 μm. Numbers in the corners are mean ADP-ribose intensity in EdU positive nuclei normalized to WT sample, quantified in ImageJ.

(B) Representative images from the data quantified in Figure 2D of levels of ADP-ribose and PCNA immunostaining in *MSH3*/*MLH1*-deficient HCT116 cells and in their Chr3 complemented (*MLH1*) or CHr3 & Chr5 complemented (*MLH1* & *MLH3*) derivatives, following incubation for 60 min with PARGi. Scale bar, 20 μm.

(C) Representative ScanR images from the experiment in Figure 2E showing ADP-ribose levels in PCNA-positive $Rnaseh2b^{+/+}$ and $Rnaseh2b^{-/-}$ MEFs following incubation with PARGi for 60 min (*left*). RNase H2 immunoblotting (*bottom right*) and cell extract activity assays (*top right*) confirming the absence of residual Rnaseh2 in $Rnaseh2b^{-/-}$ MEFs. Note that the band migrating as 18-mer product in reactions containing Rnaseh2-deficient extracts is contaminant present in the substrate preparation (see lane 1).

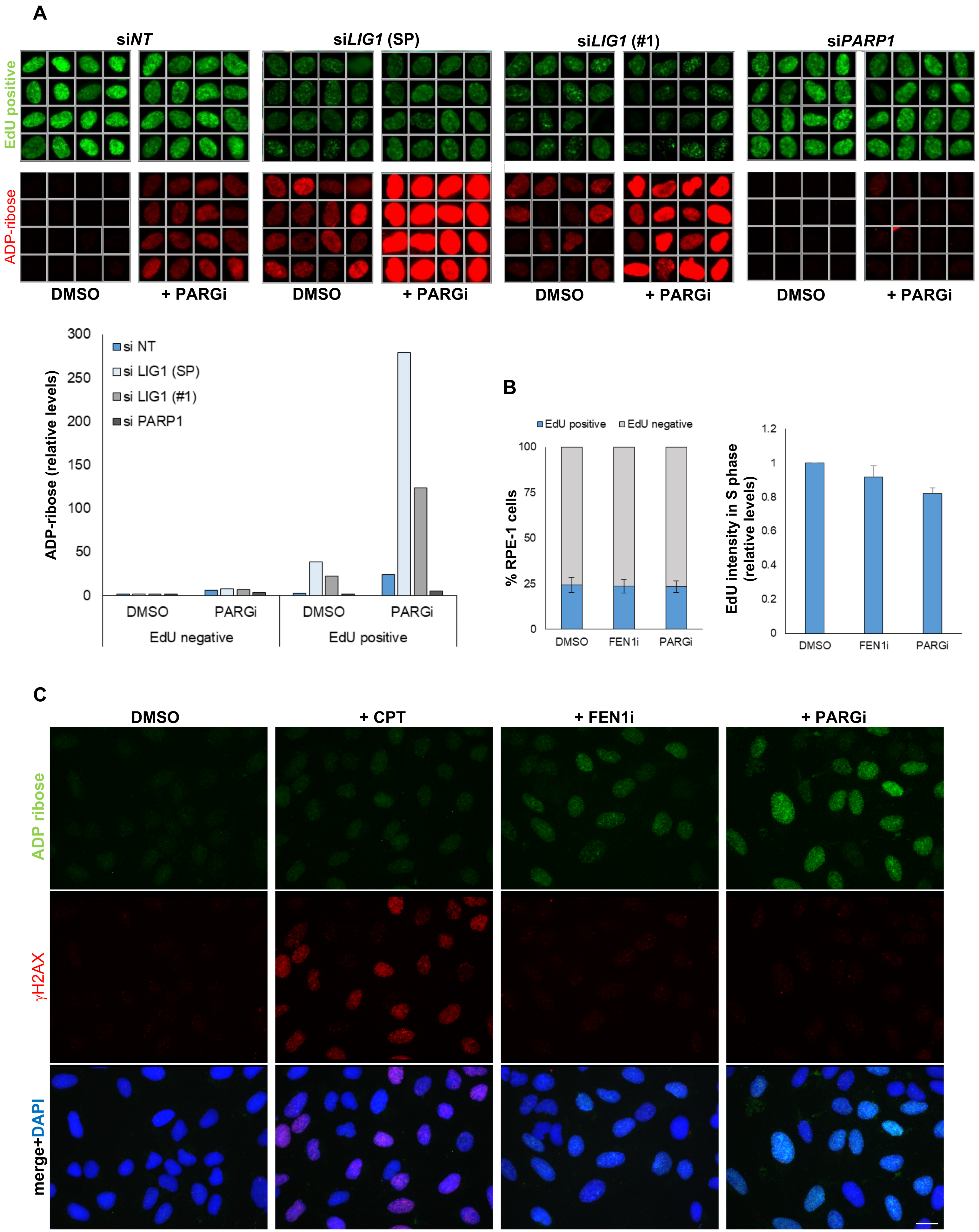
A
siNT
siLIG1 (SP)
siLIG1 (#1)
siPARP1
EdU positive
ADP-ribose
DMSO
+ PARGi
DMSO
+ PARGi
DMSO
+ PARGi
DMSO
+ PARGi
si NT
si LIG1 (SP)
si LIG1 (#1)
si PARP1
ADP-ribose (relative levels)
300
250
200
150
100
50
0
DMSO
PARGi
DMSO
PARGi
EdU negative
EdU positive
B
EdU positive
EdU negative
% RPE-1 cells
100
75
50
25
0
DMSO
FEN1i
PARGi
EdU intensity in S phase (relative levels)
1.2
1
0.8
0.6
0.4
0.2
0
DMSO
FEN1i
PARGi
C
DMSO
+ CPT
+ FEN1i
+ PARGi
ADP ribose
γH2AX
merge+DAPI

Figure S3. S phase poly(ADP-ribose) levels are increased in LIG1-depleted cells (related to Figure 3).

(A) Representative ScanR images (*left*) and quantification (*right*) of ADP-ribose in RPE-1 cells transfected with the indicated siRNAs. 72 hr after transfection, cells were incubated for 20 min with 10 μM EdU in the absence or presence of PARGi. For representative images only the EdU positive cells are shown, for quantification the EdU-negative (non-S phase) and EdU-positive (S phase) cells were gated according to nuclear EdU intensity. Data are from a single experiment.

(**B**) ScanR quantification of EdU positive vs. negative RPE-1 after 20 min incubation with DMSO vehicle, FEN1i or PARGi together with EdU (average of n=3 with SEM, *left*). Mean intensity of EdU in similarly treated EdU positive cells (average of n=3 with SEM, *right*).

(C) Indirect immunofluorescence imaging of ADP-ribose and γH2AX in RPE-1 cells after incubation for 30 min with DMSO vehicle, CPT, FENi or PARGi. Scale bar, 20 μm.

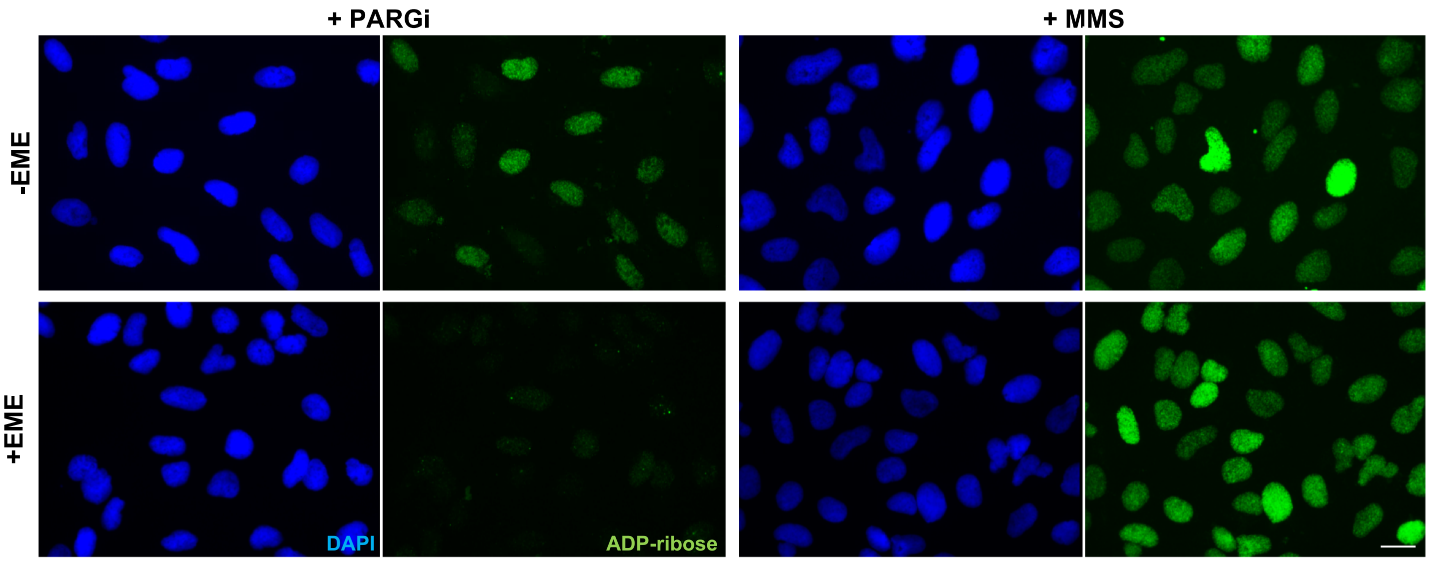

Figure S4. Suppression of Okazaki fragment formation with emetine prevents S phase ADP-ribosylation (related to Figure 4).

U2OS cells were incubated or not with emetine (EME) for 45 min as indicated, with PARGi or MMS added during the final 20 min. The cells were then pre-extracted, fixed and stained with ADP-ribose antibody. Scale bar, 20 μm.

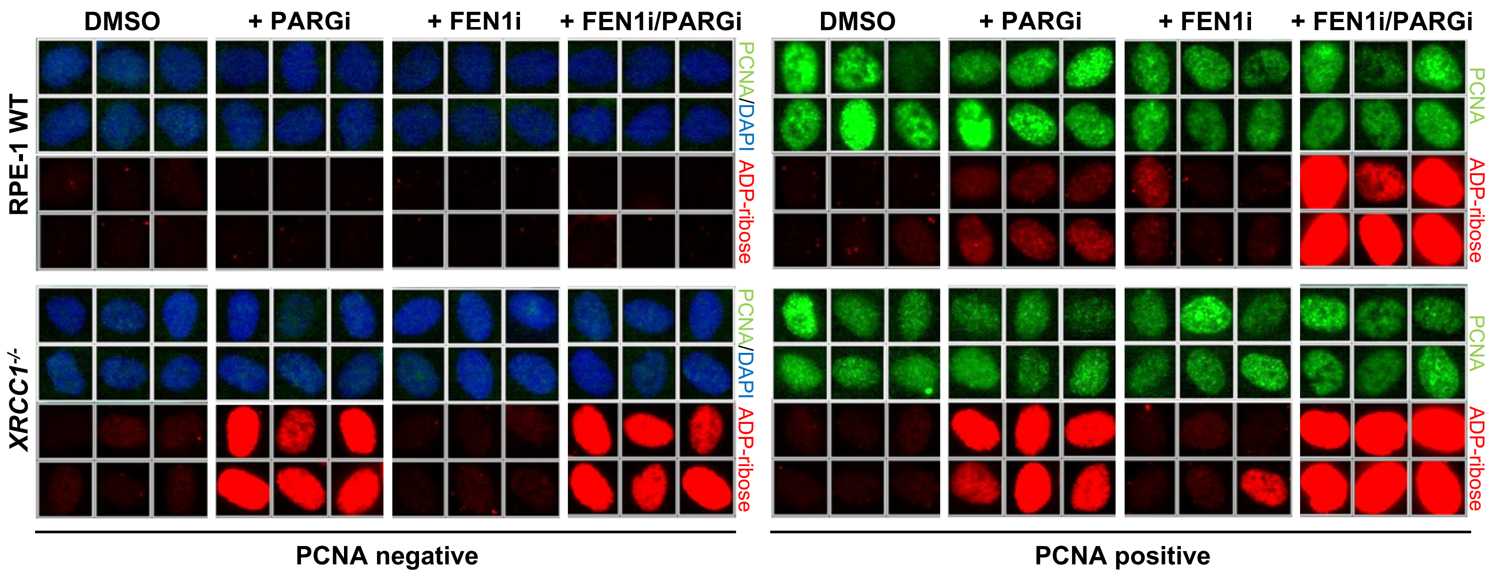

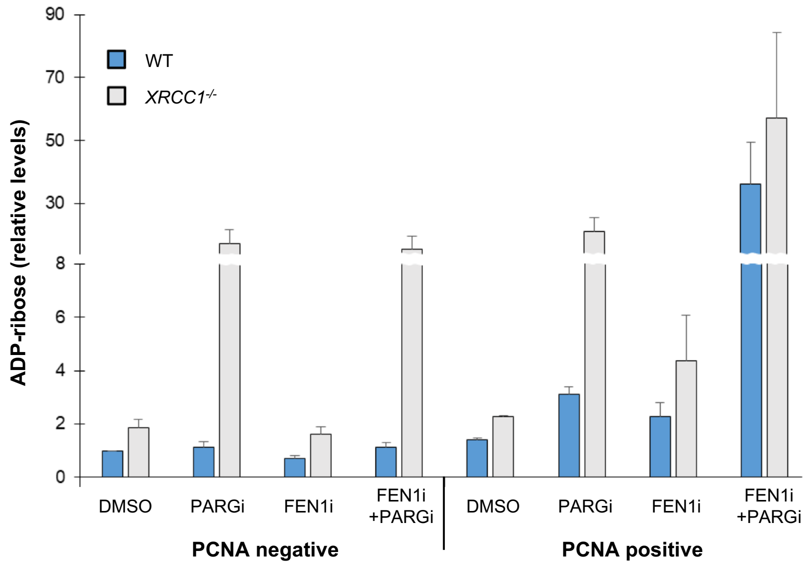

Figure S5. Elevated poly(ADP-ribosyl)ation in *XRCC1*$^{-/-}$ cells outside of S phase (related to Figure 5). Representative ScanR images and quantification of ADP-ribose by ScanR imaging in wild type (WT) and *XRCC1*$^{-/-}$ RPE-1 clonal cell lines. Cells were treated with DMSO vehicle or FEN1i for 30 min, with PARGi added or not during the last 15 min, as indicated. Note the break and change in scale in the Y-axis required to display the very high ADP-ribose levels induced in *XRCC1*$^{-/-}$ RPE-1 cells (average of n=3 with SEM).
